# Long-term evolution of fish communities in European mountainous rivers: past log driving effects, river management and species introduction (Salzach River, Danube)

**DOI:** 10.1007/s00027-015-0398-3

**Published:** 2015-06-05

**Authors:** Gertrud Haidvogl, Didier Pont, Horst Dolak, Severin Hohensinner

**Affiliations:** Institute of Hydrobiology and Aquatic Ecosystem Management, University of Natural Resources and Life Sciences Vienna, Max Emanuelstrasse 17, 1180 Vienna, Austria; Research Unit Hydrosystems and Bioprocesses, IRSTEA, Antony, France

**Keywords:** Salzach catchment, Alpine rivers, Historical ecology, Fish community changes, Log driving

## Abstract

Using historical sources from the turn of the 19th to the 20th century, we investigated the long-term evolution of the fish community in a mountainous river network and the influence of different human uses and management measures. Within the alpine Salzach catchment, historical presence was reconstructed for 26 fish species, abundance classes for 19 species. Due to channelization, flood protection and dam erections, the spatial distribution of fish species was reduced during the 20th century. Many rheophilic and eurytopic fish species historically inhabited river reaches along a wide longitudinal profile and were present in more upstream river reaches than nowadays. The decrease of species diversity in the headwater sections is a consequence of lost lateral connectivity. Strongest effects are reported for sensitive species requiring different habitat types during their life cycles (especially pike, nase, Danube salmon). One of the most important shifts from the historical fish community to the present one reflects the deliberate introduction of fish species for fisheries. Rainbow trout and brook trout, absent from the historical fish assemblage, today represent up to 29 % of the total number of fish occurrences. In contrast, log driving, one of the most common historical pressures in European mountainous rivers, did not show significant negative effects on the past fish ecological situation. This result strongly differs from the impacts of log driving and deforestation demonstrated for recent times, and could be related to the change in log driving practices during the 20th century and to the high societal value of fish before the industrialization period along with other historical pressures affecting fish in rivers without log driving. In general, our results can be valid for a large number of European mountainous rivers. They highlight the usefulness of such detailed historical studies for our understanding of the long-term evolution of fish communities and their present functioning, and point the way for future river management strategies to restore fish biodiversity.

## Introduction

Rivers and their fish communities have been modified by humans for millennia to operate mills, to serve as transport routes and recipients of waste, or to harvest aquatic animals and plants. The societal impact increased since the onset of industrialization in the late 18th century. Technological inventions and a new concept of the human-nature relation went hand in hand with a shift from solar-based to fossil energy sources (Fischer-Kowalski and Haberl 2007). This resulted in an unprecedented, systematic and large-scale exploitation of natural resources along with accelerated exchange of materials and goods, as well as in new technical means to control natural processes. Considering the consequences of this development on rivers, Jakobsson (2002) introduced the term “industrialization of rivers”. Human uses of rivers intensified in the 19th and 20th century and they were progressively supported by new technical infrastructures and political programs (see e.g. White 1995 for an early study of the Columbia River or Castonguay and Evenden 2012; Mauch and Zeller 2008 for recent compilations about European and North American rivers). On large and medium-sized rivers, systematic channelization measures improved shipping to meet the requirements of steam ships, which started to replace wooden ships after ca. 1810. Artificial shipping channels connected river catchments and enabled biological invasions. Systematic flood protection measures started mainly in the late 19th century as a result of demographic growth and the subsequent spread of settlements and agriculture towards floodplains (Blackbourn 2007; Haidvogl 2008; Haidvogl et al. 2013). Hydropower dams altered fluvial hydromorphology and habitats in particular in the 20th century (e.g. Evenden 2004). Moreover, organic and non-organic pollution intensified (e.g. Cioc 2002 for the Rhine). These developments were accompanied by the continued use of rivers for floating of timber and fuel wood, which affected aquatic species during the wood transport itself but also because of habitat change due to maintenance work during the year (Gingrich et al. 2012). In pre-industrial times and during the transition phase to the industrial period, fuel wood was the main energy source and local and regional supply was indispensable for private households, local crafts or mining. In the 20th century, log driving and wood floating was mainly done to supply large industries (saw mills, pulp and paper factories). In addition, biological interventions gained new momentum in the second half of the 19th century because improved transport facilities and progress in artificial reproduction technology promoted the introduction of alien fish species (e.g. Halverson 2012 for rainbow trout).

The described human interventions altered riverine fish communities. The current estimate is that between 40 and 80 % of species are now imperiled in Europe; in North America the percentage is between 27 and 35 % (Helfman 2007; Kottelat and Freyhof 2007; Tockner et al. 2009). Habitat change including pollution, exploitation and species introductions are considered as important drivers (Helfman 2007).

Reconstructing historical fish ecological conditions can highlight biodiversity changes and species decline as well as the associated temporal trends on the catchment or single river scale. Such studies can serve as a reference for ecological assessment, as required in Europe for example by the EU-Water Framework Directive (European Commission 2000), and as a basis for planning river restoration measures. Furthermore, historical reconstructions help investigate the fish ecological impacts of human alterations such as habitat change, (over-)exploitation along with intentional species introduction or unintended dislocation of species.

Carrel (2002), Wolter et al. (2005) or Winter et al. (2009) reconstructed past fish communities and relative abundance of the French Rhone, the Lower Elbe and Oder Rivers and the Dutch Vecht River as a basis for comparison with present conditions. Hamilton et al. (2005) analyzed how dams erected in the Upper Klamath River basin since the 1910s changed the occurrence of diadromous fish. Koel and Sparks (2002) investigated the relation between various discharge parameters and the abundance of age-zero fish in the Illinois River since the late 1950s. Rinne et al. (2005) addressed the relation between fish composition changes and social causes in North America and analyzed for instance the interactions between land use, related stream nutrient concentrations along with in-channel degradations triggered by damming, channelization, pollution or biological alterations. Long-term anthropogenic influences and subsequent changes in freshwater fish populations over centuries have been demonstrated as well (Humphries and Winemiller 2009). Mill dams affected migration and exploitation of diadromous fish as long ago as in the Middle Ages (Hoffmann 1996). Carp breeding and trading, for example, has led to the unintended dispersal of other fish species (see e.g. Van Damme et al. 2007).

Alpine gravel-bed rivers have been subject to a succession of human interventions especially since the last two centuries. Often, originally braided rivers were channelized and straightened, and the specific ecological functions characterizing such systems were altered, including high dynamic and bed load transport and the strong connection to the surrounding terrestrial zone. This impacted the specific cold-water preferring fish community (Piégay et al. 2009; Pont et al. 2009). Habitat change and biological alterations were intertwined with the effects of long-term climate variability (Brázdil et al. 2006, 2010 for an overview of Europe). It is expected that future climate change will cause warm-water preferring species to expand their spatial range and cold-water preferring species to shift upward in elevation to colonize suitable habitats, e.g. in Alpine catchments (Comte and Grenouillet 2013).

This study describes the history of the Salzach river system, a tributary of the Inn, which is itself the largest tributary of the Upper Danube, from the late 19th century until the present time. While existing studies analyze mainly single rivers and fish composition changes on a large scale, our investigation focuses on a whole catchment and employs a spatial analysis of historical fish species distribution on morphologically homogeneous river segments. This allows studying quantitative changes in terms of species distribution and abundance classes. In detail, we investigated (1) fish communities of the main river and its tributaries around the turn from the 19th to the 20th century, (2) the long-term (= longitudinal) evolution of the fish community until the present time, (3) the upstream–downstream distribution of the fish species in the historical Salzach catchment compared to the present situation in comparable Austrian rivers and (4) the effects of log driving on fish around 1900.

## Materials and methods

### Historical fish data

**Fig. 1:**
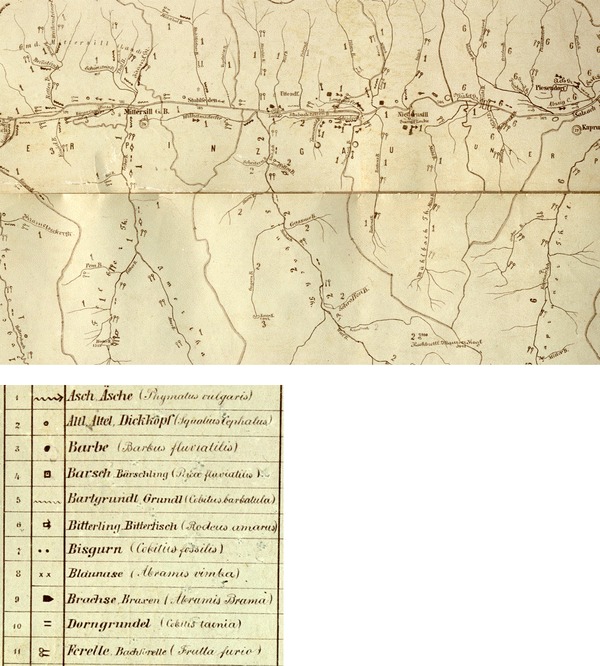
Detail of Kollmann’s fish distribution map: example of the Upper Salzach with the tributaries Felber Ache und Stubache. Presence of single fish species was indicated using species *specific symbols*. Some examples are shown below based on a section of the original legend. *Äsch* grayling, *Altl* chub, *Barbe* barbel, *Barsch* Perch, *Bartgrundl* stone loach, *Bitterling* bitterling, *Bisgurn* weather fish, *Blaunase* vimba, *Brachse* bream, *Dorngrundel* spined loach, *Forelle* brown trout

**Fig. 2:**
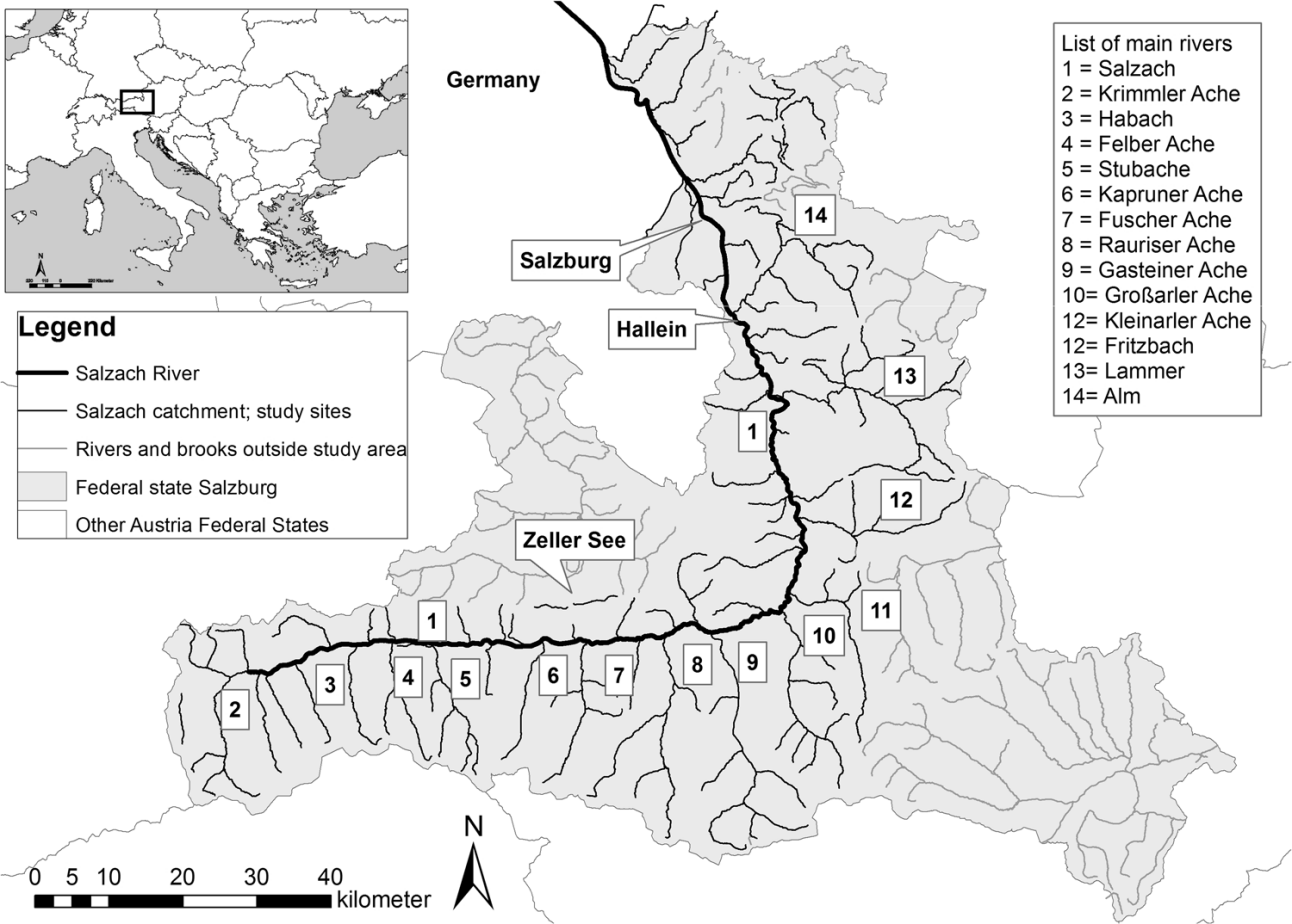
The Salzach River and its main tributaries in the Austrian Federal State of Salzburg considered in this study; only rivers with a catchment >10 km^2^ shown. *Numbers* referring to tributaries listed on the *top right* are always placed on the *right side*

Historical fish data were taken from a fish distribution map from 1898 (Kollmann 1898; Fig. 1) and the fishery cadastre of Salzburg (1904). Kollmann’s map considers 38 species (including lamprey), of which 26 occurred in the Salzach catchment studied here (Fig. 2). The map was based on questionnaires completed by municipalities and fishing right owners who reported information about fish species occurring in the river sections under their jurisdiction. Kollmann provided a list of fish species indicated by vernacular names and the corresponding scientific names. It was not possible to identify the taxonomy used, because the scientific names do not always match with common systems of that time (e.g. Heckel and Kner 1858 or Siebold 1863). For our analyses, species were assigned based on the species names given by Kollmann according to the taxonomy of Kottelat and Freyhof (2007). Most species (21) could have been determined with certainty, but for some rare species the identification is not absolutely certain (bleak *Alburnus alburnus*, Danubian bream *Ballerus sapa*, dace *Leuciscus leuciscus*, perlfish *Rutilus meidingeri* and vimba *Vimba vimba*). The weatherfish (*Misgurnus fossilis*) may also have been reported incorrectly by fishermen and local officials. The spatial resolution of the map is very detailed: fish species are also given for small rivers and headwaters. For each fish point in the map, a point was recorded in ArcGIS. Then, this point-layer was intersected with the digital river network, yielding species presence on the segment level. Segments represent hydro-morphologically homogeneous river sections (see also below). The slight mismatch of Kollmann´s delineation of segments based on municipalities and fishing rights and that of the digital river network may be a source of spatial inaccuracy. Accordingly, a fish species could have also occurred in the next segment up- or downstream.

**Fig. 3:**
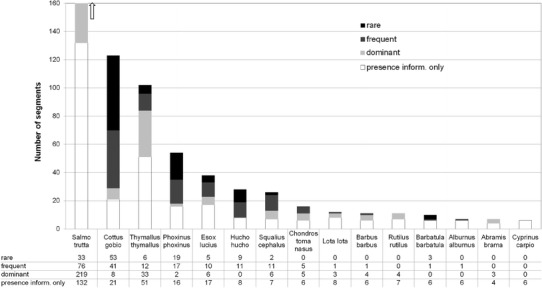
Historical presence and abundance of fish species per segment (total number of segments with presence information = 547; total number of segments with abundance information = 382, only species with presence in more than 5 segments shown)

The 26 fish species mentioned in the distribution map of Kollmann occur in 547 segments. Brown trout was the most frequent fish with a presence in 460 or 84 % of the segments (Fig. 3). The second species was the bullhead, which occurs in about one-fifth of the segments. Grayling was present in 18 %, the European minnow in about 10 %. All other species were found in 50 segments or fewer, i.e. they occurred at a maximum of ~7 % of the river segments.

The fishery cadastre, in contrast to Kollmann´s map, considers only 19 species important from a fishery perspective, among them brown trout (*Salmo trutta*), grayling (*Thymallus thymallus*), Danube Salmon (*Hucho hucho*), pike (*Esox lucius*), nase (*Chondrostoma nasus*), barbel (*Barbus barbus*), European minnow (*Phoxinus phoxinus*), chub (*Squalius cephalus*), bullhead (*Cottus gobio*) and stone loach (*Barbatula barbatula*). Besides the Salzach, only larger or medium-sized tributaries were considered; information is lacking for small brooks. The cadaster is a valuable amendment of Kollmann´s map because it reports the abundance verbally. This information was transferred into the three classes dominant, frequent and rare. The abundance class was added in the GIS-layer to the fish points identified based on the Kollmann map.

Abundance information was available from the fisheries cadastre for a total of 382 segments (Fig. 3). Similar to species presence, most information refers to trout (abundance class available for 328 segments out of 460 with presence information for trout), bullhead (102 out of 123 segments) and grayling (51 out of 102 segments). Among the 19 species for which the abundance classes were given, there were eleven for which details were available for only 5 or even fewer segments.

Compared to other historical sources of the late 19th and early 20th century (for instance fish biological descriptions, anecdotal reports), Kollmann´s map and the fishery cadastre are exceptionally detailed and accurate. As for all historical sources, however, some limitations have to be accepted. Both sources focus more on commercially important fish species, and small or rare fish might be incomplete or missing entirely as in the cadastre. Furthermore, we cannot be certain about the correct identification of rare species by fishermen. Abundance information was not standardized; responses were certainly based on personal evaluations and must be taken as relative estimates (see Haidvogl et al. 2014 for use of historical sources). In a recent study, Pont et al. (2015) compared the historical fish occurrences derived from Kollmann’s map with predictions from 21 species distribution models (SDM) calibrated with present electro-fishing samplings (Logez et al. 2012). The regressions of the 14 species-specific observed values (historical data) and the corresponding expected values (modeled data) were highly significant highlighting the good predictive performance of the SDM and the good quality of the historical data from the Salzach catchment, at least for the most common species for which SDMs were available.

### Present fish data

We used two datasets with present fish samples, all collected by electro fishing. One covered the Salzach catchment, where fish surveys were available for only a few rivers or sections. The data stem mainly from recent monitoring campaigns for the EU Water Framework Directive (WFD); some samples were taken from the Salzach during specific projects and date back to 1992. To compare historical and present data on abundance levels, we transformed the individuals/ha values available for contemporary samples into the three abundance classes used for the fishery cadastre. The thresholds for the three classes depended on the total number of species at a site (1–14 species) and ranged from 12 to 100 % for class dominant, 5–60 % for class abundant and 0.01–12 % for class rare.

Since present fish samples were available only for 45 segments of the Salzach catchment, we obtained further data for Austria as a whole from the Austrian database for the WFD. These samples were taken from river segments with similar environmental conditions as the historical Salzach fish-dataset and we used them to compare the relative occurrence of fish species along longitudinal gradients.

### Environmental variables

For the segments of the digital river network (see below), several environmental variables were computed: The altitude of the start and end point of each segment was derived by intersecting each of these points with a digital terrain model of Austria (resolution 10 × 10 m). Then, the slope was computed based on the two elevation measures and the length of the river segment. The distance to the source was calculated as the total sum of all segments upstream; the size of the catchment upstream of a segment was obtained from the catchment characterisation and modelling CCM-river network (De Jager and Vogt 2010). The latter was a representative variable for river size because no digital information on the mean discharge in the segments was available. Fish regions were available for segments with a catchments size >10 km^2^. For each segment, historical annual mean air temperature values were added from the HISTALP project (http://www.zamg.ac.at/histalp/component/option,com_frontpage/Itemid,1/index.html, download December 2011; period from 1890 to 1900; see Chimani et al. 2013 for details).

All these environmental data used for the investigation of the historical conditions of the Salzach catchment were also available for present fish data of Austria as a whole.

### Human uses

**Fig. 4:**
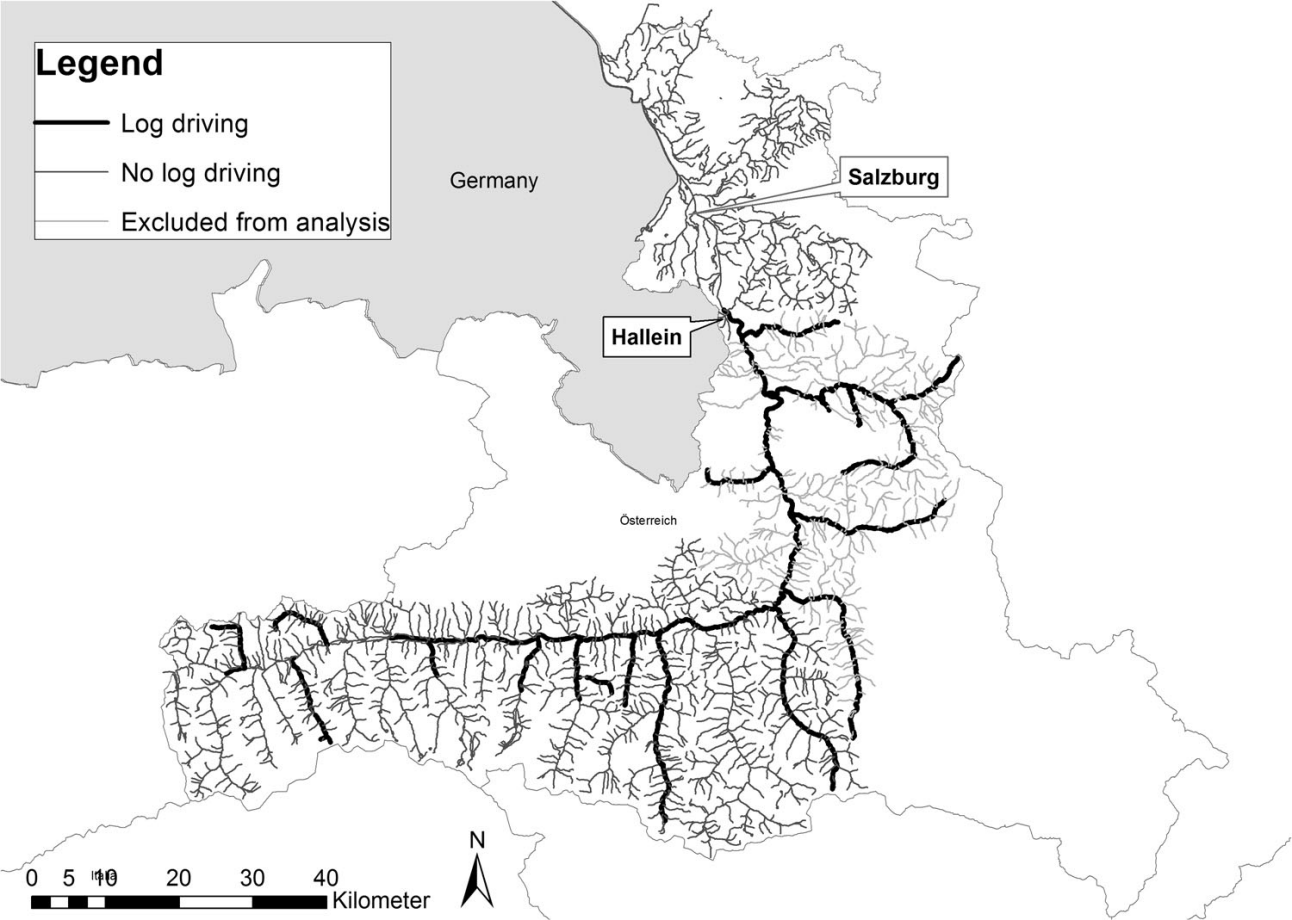
Rivers used for log driving in the late 19th century; tributaries of the middle Salzach catchment shown in *light grey* were excluded from the analyses (see text for explanations)

We incorporated log driving as a main historical river use. Sources documenting this activity in the Salzach catchment are available since the 16th century although the practice it certainly older. For the late 19th century it was explicitly mentioned in Koller (1975) for 102 segments (see Fig. 4). This is, however, a minimum number because smaller tributaries of the main log driving rivers no doubt also fulfilled this purpose but were not mentioned. Log driving required complex management and coordination. From an ecological point of view two activities were relevant. First, log driving itself, which was in the Salzach catchment usually done in late spring with second half of April and May as the main months. By that time the ice cover on the brooks had melted and the discharge increased due to the snow melt. The sequence of logging in the different tributaries had to be coordinated in order to ensure that the number of logs could be handled at the main rack in Hallein. Only on few larger rivers such as the Salzach itself was logging done without artificial freshets. On most brooks, splash dams were built at narrows or gorges. During the log driving period the reservoir upstream of a splash dam was filled. Afterwards, gates in the dam were opened and the wood was flushed downstream. According to a report from 1823 this was done e.g. on the Taugl twice a day over a period of about 4 weeks per year (Koller 1975). At this time aquatic organisms experienced several hours of very low residual flow each day when the reservoirs were filled, as reported e.g. for the Salza, a tributary of the Enns (Vogel 1987). This alternated with sudden water flushes which were mixed with the logs and mobilized sediment. The second activity was the maintenance and preparation of the rivers for log driving. Trees and larger rocks were usually removed as they were potential obstacles for the smooth driving. This changed the habitats of fish and other organisms even outside of the actual log driving period.

Information compiled from Koller (1975) was added to the river segments in ArcGIS. We classified (a) river segments for which we were confident that they were used for log driving in 1900 and (b) rivers for which we knew only that they were used for floating in 1880 but their status in 1900 was unclear. Most wood was floated to the salt mine and salt processing facilities in Hallein. Many small brooks of the Salzach tributaries between Hallein and Kleinarler Ache were certainly used for wood transport but are not specifically mentioned in the forestry literature we consulted; we therefore excluded them from the analysis (see Fig. 4). To test the historical impact of fuel-wood floating on fish, we compared species presence and abundance classes of segments with and without this human pressure.

Information about hydropower plants was available from “Wasserbuch des Landes Salzburg”, which lists all water rights granted since 1872 to individual persons, companies or associations. Four variables were introduced depending on the year when the right was obtained (plants built before 1900, between 1901 and 1950, 1951 and 2000, and after 2001). We could not systematically describe the intensity of pressure because details about dam heights or abstracted water volumes were not available for all facilities. River regulation and changed habitat conditions were available for the main river, the Salzach, from Piller (2012). Gerabek (1969) provides information about tributaries.

### Data analysis

The long-term change of the fish community within the Salzach catchment was investigated by comparing the 45 segments which had information for both time periods (historical and present). A Fisher’s exact test was used to identify differences in the presence of species, expressed as number of segments with occurrence, and a Kruskal–Wallis rank test for species abundance classes.

To describe the historical longitudinal distribution of the fish species and to compare it with the present situation, we defined a common synthetic environmental gradient (see Table 2 below). First we selected the river segments where the ranges of four environmental variables (slope, elevation, upstream catchment area, mean annual air temperature) were similar for the present and the historical dataset (145 and 172 river segments, respectively). Then, the environmental variables were combined using a principal component analysis. The first principal component integrated 58 % of the total variance and described mainly the longitudinal organization of river segments: from upstream (positive coordinates: highest elevation and slope) to downstream (negative coordinates: highest temperature and catchment area). Four classes of equal range were delineated to compute, for each fish species, the historical and present relative occurrence per class.

To investigate the effects of log driving, we compared a sub-dataset of 118 segments with and without log driving. The 59 segments selected randomly for each case had similar environmental conditions in terms of altitudes, slopes, rivers sizes (expressed here as distance to source) and temperature conditions. Differences in the presences of species were tested with a χ^2^-test, those for abundance classes with a Kruskal–Wallis rank test.

### Study site and digital network of homogeneous segments

The headwater stream of the 225-km-long Salzach River arises in the west of the Austrian Federal State Salzburg at approximately 2300 m altitude. The river crosses an alpine valley in an eastwardly direction for about 90 km before the middle and lower section turn north. The mouth into the Inn is located at about 350 m altitude. The Salzach has a total catchment size of 6734 km^2^. At the confluence to the Inn the mean annual discharge amounts to 250 m^3^ s^−1^. The largest tributary is the Saalach, which was not considered in this study because it flows partly in Bavaria and historical fish information was thus not complete. Other larger tributaries are Rauriser Ache (catchment size 262 km^2^), Gasteiner Ache (332 km^2^), Großarler Ache (237 km^2^), and Lammer (400 km^2^, see Fig. 2). The hydrology of several right-hand tributaries of the upper catchment is influenced by two glaciers, the Großvenediger and the Großglockner. The runoff regime of the upper and middle Salzach is thus nivo-glacial up to the gauging station St. Johann/Pongau, with a nival regime only over a short section downstream of the outflow of lake Zeller See (Mader et al. 1996). Today the hydrology of the Salzach is influenced by large reservoirs for energy production; in January and February the mean discharge is 50 % higher than prior to their construction (Muhar et al. 1996).

For our study we used a digital river network for which two datasets were combined: rivers with a catchment size higher than 10 km^2^ were imported from the Austrian National River Network (BMLFUW 2009). This network consists of 506 segments delineated based on hydrological and morphological characteristics and was prepared for the implementation of the EU Water Framework Directive. For these segments additional variables were available, such as river names or fish region. For small brooks with a catchment size below 10 km^2^, information was taken from the digital Austrian Topographic Map. In this map the segments do not account for hydro-morphological conditions and comprise river sections between two confluences. Nonetheless, we considered the resulting inaccuracy as acceptable because only small brooks with more or less similar conditions were concerned. Note, however, that the average slope for such segments is only a rough estimation because the river reaches in the valley bottoms with lower slopes were not distinguished from the steep headwaters. Altogether 1582 segments were added, most of them in high alpine altitudes. This yields a total of 2088 segments within the considered Salzach catchment. However, many headwaters in higher alpine altitudes are not suitable for fish. Thus only the 547 hydro-morphologically homogeneous segments with a historical presence of fish were kept for further analysis. The average length of a segment is approximately 3 km (range 100 m–27 km).

## Results

### Occurrence and abundance of fish species around 1900

The high frequency of trout and bullhead followed by grayling is characteristic for alpine river catchments with many small and medium-sized rhithral headwaters of the trout and grayling zones. Barbel and nase as typical and dominating species of the epipotamal river zone were limited to the middle and lower Salzach as well as to the lower sections of larger tributaries. These two species, however, were in the Salzach catchment more abundant only in the lower section of the main river downstream of Hallein where the fish region changes to epipotamal. If brown trout and grayling occurred in a segment, then they usually dominated the local fish community, which is again in accordance with the alpine habitat conditions. Bullhead occurred in 101 segments associated with brown trout. Its abundance was mostly reported as having been frequent or rare. For nase there were only 10 segments with abundance details, among them five where it was classified as dominating species.

### Long-term evolution of the Salzach fish community from 1900 to the present time

Present fish data were available only for the Salzach itself and for some larger tributaries (namely Blühnbach, Fischach, Fuscher Ache, Gasteiner Ache, Großarler Ache, Kleinarler Ache, Krimmler Ache, Lammer, Oichtenbach, Reitbach, Stubache, Tauglbach and Weissenbach). Among the 45 segments with present fish data, some lacked historical presence and abundance information; therefore only 41 segments were used for the comparison.

The total number of fish species increased from 21 to 23 (Table 1). Two of the species found in recent surveys—rainbow trout (*Oncorhynchus mykiss*) and brook char (*Salvelinus fontinalis*)—were deliberately introduced for fishery purposes. Today they often dominate in smaller rivers. First attempts to introduce rainbow trout were made in the late 19^th^ century. Nonetheless, according to the literary estate of Freudlsperger, brown trout was still more often stocked than rainbow trout between ca. 1910 and 1930, at least in the lower Salzach and tributaries there (see Archiv der Stadt Salzburg). The eel (*Anguilla Anguilla*) is a non-native species in the Salzach catchment and in the Danube in general. Spirlin (*Alburnoides bipunctatus*) and gudgeon (*Gobio gobio*) were not reported by Kollmann or the fishery cadastre but were mentioned for the Salzach in earlier sources (Heckel 1854; Zetter 1859, see also Haidvogl et al. 2014). We therefore assume that these species were ignored in the two historical fish information sources we used and not absent in general. The silver bream (*Blicca björkna*) was not reported in other historical sources, but Schmall and Ratschan (2011) estimate that it was a native species in the Salzach catchment; Kollmann may have confused it with the zobel (*Ballerus sapa*). The arctic char was historically present in other river segments studied here but not in those which we could use to compare the historical and the present situation. All five species which were no longer observable in the present surveys were historically rare.

**Table 1:** Species presence and abundance class in the Salzach catchment around 1900 and at present time (number of segments given; abbreviations abundance classes: A = dominating; C = common/frequent; R = rare; Z = zero which means that species was absent or present but no abundance information available; p values for species with a significant difference in bold)

Species	Species presence			Species abundance		
	Historical	Present	χ^2^ (p value)	Historical	Present	Kruskal test (p value)
Abramis brama	3	0	0.2407	A:1,C:0,R:0,Z:40		0.317
Alburnus alburnus	1	1	1		A:0,C:0,R:1,Z:40	
Alburnoides bipunctatus	0	3	0.2407		A:1,C:1,R:1,Z:38	
Anguilla anguilla	0	2	0.4938		A:0,C:0,R:2,Z:39	
Ballerus sapa	2	0	0.4938			
Barbatula barbatula	5	2	0.4321	A:0,C:1,R:3,Z:37	A:0,C:1,R:1,Z:39	0.411
Barbus barbus	8	3	0.1935	A:4,C:1,R:0,Z:36	A:1,C:1,R:1,Z:38	0.417
Blicca björkna	0	1	1		A:0,C:0,R:1,Z:40	
Chondrostoma nasus	9	2	**0.048**	A:4,C:3,R:0,Z:34	A:0,C:0,R:2,Z:39	0.061
Cottus gobio	24	27	0.6491	A:2,C:11,R:10,Z:18	A:2,C:4,R:21,Z:14	0.863
Cyprinus carpio	1	1	1		A:0,C:0,R:1,Z:40	
Esox lucius	14	3	**0.0053**	A:1,C:7,R:4,Z:29	A:0,C:0,R:3,Z:38	**0.007**
Gobio gobio	0	2	0.4938		A:0,C:0,R:2,Z:39	
Hucho hucho	13	2	**0.0032**	A:0,C:8,R:5,Z:28	A:0,C:0,R:2,Z:39	**0.001**
Eudontomyzon mariae	1	0	1			
Leuciscus leuciscus	2	3	1		A:0,C:0,R:3,Z:38	
Lota lota	2	0	0.4938	A:1,C:0,R:0,Z:40		0.317
Oncorhynchus mykiss	0	29	0	A:0,C:0,R:0,Z:41	A:1,C:8,R:20,Z:12	0
Perca fluviatilis	1	3	0.6156	A:0,C:1,R:0,Z:40	A:0,C:0,R:3,Z:38	0.326
Phoxinus phoxinus	11	6	0.2758	A:1,C:3,R:7,Z:30	A:0,C:0,R:6,Z:35	0.132
Rutilus meidingeri	2	0	0.4938	A:0,C:0,R:1,Z:40		0.317
Rutilus rutilus	2	6	0.264	A:1,C:0,R:0,Z:40	A:0,C:0,R:6,Z:35	0.057
Salmo trutta	35	40	0.1088	A:31,C:0,R:1,Z:9	A:23,C:7,R:10,Z:1	0.352
Salvelinus fontinalis	0	25	0	A:0,C:0,R:0,Z:41	A:2,C:0,R:23,Z:16	0
Salvelinus umbla	0	2	0.4938		A:0,C:0,R:2,Z:39	
Squalius cephalus	13	5	0.06	A:3,C:7,R:2,Z:29	A:0,C:2,R:3,Z:36	**0.037**
Thymallus thymallus	15	15	1	A:13,C:0,R:0,Z:28	A:1,C:3,R:11,Z:26	0.581
Tinca tinca	1	1	1		A:0,C:0,R:1,Z:40	
Total number of species	21	23		16	23	

The number of segments with the presence of nase, pike and Danube salmon decreased significantly. In the recent surveys they occur only in a fifth of the segments (nase and pike) or declined even to 15 % of their historical distribution. These three species are sensitive to hydromorphological habitat changes induced by channelization, damming and lateral disconnection. The distribution of chub also declined markedly. The results on occurrence were supported by our species abundance data, in particular for pike, Danube salmon and chub; for nase and roach the differences were close to significant (Table 1).

### Historical longitudinal distribution of fish species in the Salzach catchment and present situation in Austrian rivers

The four river size classes built based on the synthetic variable encompassed a longitudinal gradient with a mean elevation from 462 to 1019 m.a.s.l, a mean slope from 5 to 72 ‰, a mean catchment size from 717 to 37 km^2^ and a mean annual air temperature from 7 to 4 °C for the historical dataset (Table 2). The ranges of environmental variables associated with each of the four river size classes are quite large in particular for the smaller rivers (classes 3 and 4). The latter comprise alpine headwaters but also the small Salzach tributaries whose sources are in or close to the main valley floor. Low slopes and current velocities make especially the lowest sections of these brooks suitable for more fish species than the typical alpine brooks.

**Table 2:** Relative occurrence of fish species in the historical Salzach catchment (left part) and in comparable modern Austrian rivers (right part) along the longitudinal gradient (upstream: class 4; downstream: class 1); minimum and maximal values of the class given between brackets in the head line; values give the percentage of species occurrence in the segments of a class

	Historical Salzach catchment				Present Austrian rivers			
	Class 1	Class 2	Class 3	Class 4	Class 1	Class 2	Class 3	Class 4
	(−3.35 to 1.1]	(−1.1 to 0178]	(−0.178 to 1]	(1 to 4.59]	(−3.35 to 1.1]	(−1.to 0.178]	(−0.178 to 1]	(1 to 4.59]
No. of segments	26	22	39	58	53	57	41	21
Mean elevation (m.a.s.l.)	462	600	772	1019	434	587	792	1145
Min elevation (m.a.s.l.)	326	419	540	613	328	444	441	610
Max elevation (m.a.s.l.)	680	851	984	1583	750	740	1032	1677
Mean slope (‰)	5	19	31	72	5	13	17	41
Min slope (‰)	1	1	1	1	1	1	2	2
Max slope (‰)	15	55	107	136	33	48	80	138
Mean catchment size (km^2^)	717	148	88	37	600	202	176	81
Min catchment size (km^2^)	4	4	3	3	24	3	6	4
Max catchment size (km^2^)	3059	946	273	266	3053	1355	1339	399
Mean temperature (°C)	7	7	6	4	7	6	5	5
Min temperature (°C)	5	5	4	2	6	6	4	2
Max temperature (°C)	8	8	7	7	8	7	7	6
Abramis brama	11.5	9.1	0.0	0.0	1.9	0.0	0.0	0.0
Alburnus alburnus	15.4	9.1	0.0	0.0	15.1	0.0	0.0	0.0
Ballerus sapa	3.8	4.5	0.0	0.0				
Barbatula barbatula	15.4	9.1	5.1	0.0	35.8	5.3	0.0	0.0
Barbus barbus	30.8	9.1	0.0	0.0	28.3	0.0	0.0	0.0
Chondrostoma nasus	34.6	18.2	0.0	0.0	17.0	0.0	0.0	0.0
Coregonus sp.	3.8	0.0	0.0	0.0	0.0	0.0	0.0	0.0
Cottus gobio	50.0	50.0	53.8	31.0	69.8	80.7	51.2	33.3
Cyprinus carpio	3.8	9.1	0.0	0.0	5.7	0.0	0.0	0.0
Esox lucius	46.2	22.7	10.3	3.4	11.3	0.0	2.4	0.0
Eudontomyzon mariae	3.8	0.0	0.0	0.0	5.7	3.5	2.4	4.8
Hucho hucho	38.5	13.6	5.1	5.2	3.8	0.0	0.0	0.0
Leuciscus leuciscus	3.8	9.1	0.0	0.0	17.0	3.5	0.0	0.0
Lota lota	19.2	13.6	0.0	0.0	13.2	3.5	0.0	0.0
Misgurnus fossilis	0.0	4.5	0.0	0.0				
Oncorhynchus mykiss					67.9	54.4	61	42.9
Perca fluviatilis	11.5	4.5	0.0	0.0	18.9	1.8	0.0	0.0
Phoxinus phoxinus	34.6	18.2	28.2	13.8	30.2	0.0	0.0	0.0
Rutilus meidingeri	7.7	9.1	0.0	0.0				
Rutilus rutilus	7.7	9.1	0.0	1.7	15.1	7.0	0.0	0.0
Salmo trutta fario	76.9	72.7	97.4	98.3	94.3	100.0	100.0	100.0
Salvelinus fontinalis					15.1	17.5	22	33.3
Salvelinus umbla	0.0	0.0	0.0	0.0	0.0	1.8	0.0	0.0
Silurus glanis	3.8	0.0	2.6	0.0	0.0	0.0	0.0	0.0
Squalius cephalus	38.5	22.7	10.3	3.4	39.6	7.0	0.0	0.0
Thymallus thymallus	53.8	22.7	28.2	17.2	58.5	38.6	24.4	9.5
Tinca tinca	3.8	4.5	0.0	0.0	0.0	0.0	0.0	0.0

Brown trout inhabited all different river types, with an increasing relative occurrence in the smaller catchments. Six other species also occurred in all classes, but they showed a tendency opposed to brown trout. The relative occurrence of bullhead, pike, Danube salmon, minnow, chub and grayling tends to increase with river size. All other species were distributed in specific classes, among them nase and barbel, which had their highest relative occurrence in the largest rivers. Fisheries documents from the late 19th or early 20th centuries clearly indicate that barbel and nase inhabited mainly the lower Salzach section, whereby some individuals migrated also to the middle river reach. During the spawning period, nase migrated to tributaries of the lower Salzach, even though the spawning grounds here were usually located in the most downstream 2–4 km (Archiv der Stadt Salzburg 1929). The small rivers had a comparably high species richness around the year 1900. A total of eight species were recorded in the smallest catchments. Apart from trout and bullhead, also pike, Danube salmon, minnow and grayling were quite common species here. In particular pike was reported not only for small, epipotamal tributaries of the middle and lower Salzach. It was in addition recorded for the Upper Salzach which had, due to the low slope prior to channelization, a much lower velocity than nowadays and many floodplain water bodies. Many of the species, in particular in the downstream river sections (classes 1 and 2), occurred solely in very few segments. Tench or catfish as well as carp were reported only for the lower Salzach.

In contrast to the historical situation in the Salzach, most fish species today have a much smaller longitudinal distribution in Austrian rivers. Trout still occurs in all four classes, and even in all segments of classes two, three and four. Apart from that, however, only bullhead and grayling occur along the whole longitudinal profile. Epipotamal species such as barbel or nase but also minnow and pike are restricted to the largest and most downstream river segments. As in the Salzach catchment the non-native rainbow trout also became widely distributed in other Austrian rivers. Its relative occurrence in the first three classes exceeded 50 % (43 % in the smallest rivers). The species richness in the smallest rivers is currently much lower than historically and amounts to only four species and five including rainbow trout in contrast to eight and nine species at the turn of the 20th century.

### Log driving and its effect on fish around 1900

The comparison of species presence in rivers with and without log driving showed no significant differences for the 18 fish species in our data set. In fact, among the five species for which the χ^2^-test indicated a difference in the percentage of segments with species presence only the proportion of trout was slightly lower for rivers with log driving. In contrast, for bullhead, Danube salmon, chub and grayling the percentage of segments where these species occurred was even higher. Also the Kruskal test for the twelve species for which abundance class information was available did not exhibit a significant impact (see Table 3). Possible explanations for these results are discussed below.

**Table 3:** Comparison of segments with and without log driving (total number of segments = 59 with and 59 without); presence is given in percent of segments; for abundance of species indicated in brackets: number of cases per abundance class; A = dominating; C = common/frequent; R = rare; Z = zero, species absent or species present but no abundance information available)

Species	Species presence (% of segments)			Species abundance		
	No log driving	Log driving	χ^2^ (p value)	No log driving	Log driving	Kruskal test (p value)
Abramis brama	0	1.7	1			
Alburnus alburnus	1.7	0	1	A:0,C:1,R:0,Z:58	A:0,C:0,R:0,Z:59	0.317
Ballerus sapa	0	1.7	1			
Barbatula barbatula	1.7	1.7	1	A:0,C:0,R:1,Z:58	A:0,C:0,R:1,Z:58	1
Barbus barbus	3.4	3.4	1	A:0,C:0,R:0,Z:59	A:1,C:0,R:0,Z:58	0.317
Chondrostoma nasus	5.1	5.1	1	A:0,C:2,R:0,Z:57	A:2,C:1,R:0,Z:56	0.627
Cottus gobio	32.2	40.7	0.4444	A:0,C:13,R:4,Z:42	A:1,C:7,R:16,Z:35	0.425
Esox lucius	6.8	8.5	1	A:0,C:2,R:1,Z:56	A:0,C:3,R:1,Z:55	0.693
Hucho hucho	8.5	13.6	0.5581	A:0,C:1,R:2,Z:56	A:0,C:5,R:2,Z:51	0.165
Leuciscus leuciscus	0	1.7	1			
Lota lota	0	1.7	1			
Phoxinus phoxinus	20.3	18.6	1	A:1,C:6,R:1,Z:49	A:0,C:3,R:7,Z:49	0.83
Rutilus meidingeri	1.7	1.7	1	A:0,C:1,R:0,Z:58	A:0,C:0,R:0,Z:59	0.317
Rutilus rutilus	0	1.7	1			
Salmo trutta	84.7	78	0.4789	A:43,C:5,R:1,Z:9	A:44,C:0,R:0,Z:14	0.924
Squalius cephalus	5.1	10.2	0.4903	A:0,C:1,R:1,Z:57	A:1,C:5,R:0,Z:53	0.133
Thymallus thymallus	22	28.8	0.5264	A:7,C:3,R:0,Z:48	A:9,C:5,R:0,Z:43	0.355
Tinca tinca	0	1.7	1			

## Discussion

### The historical Salzach fish community and the quality of our historical data

The fish species richness of the Salzach catchment was, at the turn from the 19th to the 20th century, quite high considering the alpine environmental conditions. Clearly, many species reported by Kollmann had a limited distribution, either because they are typical lake inhabitants such as coregonids, perlfish or arctic char, or because they are adapted to more epipotamal habitat conditions which prevailed only in the lower Salzach. The fish community was clearly dominated by trout and other fish typical for rhithral brooks such as bullhead, grayling or minnow (see Noble et al. 2007 for habitat requirements of European fish species). Other historical sources reported an even higher fish species richness. Schmall and Ratschan (2011) assume a total of 39 native species and a further six species for which the written historical sources are ambiguous. Among these are zobel and perlfish, which appeared also in Kollmann’s map, whereas gibel carp (*Carassius gibelio*), moderlieschen (*Leucaspius delineatus*), bitterling (*Rhodeus amarus*) and schraetzer (*Gymnocephalus schraetzer*) were not mentioned there.

Despite these missing species the map of Kollmann and the fishery cadastre provide exceptionally detailed, reliable and consistent data compared to other historical sources from the late 19th century. The fishermen who responded to the questionnaires were certainly familiar with the fish community of their rivers and there was no reason to provide wrong information, for example because of potential problems with taxation since no information about average catch was requested. Of course, there are some limitations such as misclassification of rare species or the lack of absolute abundance information. Particularly the latter, however, has mostly become available only since standardized electro-fishing was implemented in the second half of the 20th century. At that time many European rivers were already affected by large scale habitat alterations and such information can thus not fully reflect the changes triggered by industrialisation. We decided to overcome the problem of missing absolute abundance information by transferring the historical as well as present abundance into three comparable classes. Nevertheless the comparison of historical and present abundance information was likely affected by the different sampling techniques, i.e. reports by professional fishermen (reflecting catches throughout the year) and electro fishing (representative but temporally selected surveys). In particular, bullhead was probably underrepresented in samples from the present (Reyjol et al. 2005).

### Long-term evolution of the Salzach fish community

The three species most affected by human uses in the 20th century were Danube salmon, pike and nase. The Danube salmon is a rheophilic and potamodromous species which needs gravel for reproduction, whereas the pike is eurytopic and spawns on plants of inundated floodplains. But both species are piscivorous. Similar to the Danube salmon the nase prefers fast-flowing areas and is a lithophilic spawner that undertakes spawning runs but feeds as an adult on benthic algae (Jungwirth et al. 2003; Reckendorfer et al. 2001). The decrease in both the occurrence and abundance of these three species therefore points to different human alterations of the main river, the Salzach, and the lower reaches of larger tributaries. Local river engineering measures started there in the 14th century and were continued in the following centuries. In the second half of the 19th century, river regulation intensified, initially aiming at channel stabilization. Flood protection and hydropower dams were established mainly in the 20th century (Piller 2012; Rohr 2007). Within the investigated catchment, 52 hydropower stations existed in 1900, most of them in small and medium-sized tributaries. By 1950, a further 98 dams were erected, and 129 between 1951 and 2000. Today the total number of dams is 309. These numbers show that the Salzach river network around 1900 was already subject to various hydro-morphological changes and that these modifications increased throughout the 20th century as was characteristic for many European and alpine rivers (Piégay et al. 2009; Pont et al. 2009). Dams interrupted the spawning runs of Danube salmon and nase, and dykes blocked the lateral connectivity and disconnected the floodplains necessary for pike reproduction. These structures also reduced the habitat variability in the main channel. The strong effects of these measures were also visible in the fact that even chub declined, a eurytopic species with plastic biological requirements (Noble et al. 2007). Although the Kruskal–Wallis test was not sensitive enough to reveal a significant difference for the decline of grayling, our data indicated a stronger change in the abundance classes from dominating in 13 segments around 1900 to only one today. Also, bullhead was common historically in eleven segments but only in four at present.

One of the most important changes in the fish communities is due to the introduction of rainbow trout and brook trout. These two species represent currently 29.3 % of the total number of species occurrences. A shift from brown trout to rainbow trout occurred in several small and medium-sized rivers. Archive material indicates that stocking of these species was mainly a phenomenon of the second half of the 20th century (unpublished data from Archiv der Stadt Salzburg, estate Freudlsperger; Archiv der Pfenninger Stiftung). These conclusions agree with the statement Welcomme (1988) made on the global scale.

### Historical longitudinal distribution of fish species

The comparison of the longitudinal distribution of fish species amplifies the results of the long-term changes described above. In the historical Salzach catchment, several fish species inhabited river sections along the whole longitudinal profile. Small brooks and Salzach tributaries in lower altitudes for instance offered adequate habitats not only for brown trout or bullhead but also for pike, Danube salmon, minnow or chub. The present fish communities of Austrian rivers are affected by large-scale and systematic human modifications made during the 20th century. The most important are hydro-morphological changes due to channelization, flood protection and hydropower production. Our comparison of the fish distribution along a longitudinal gradient highlights the consequences of the disturbed longitudinal and lateral connectivity. The latter, particularly in spring and early summer, is important in both braided and meandering rivers, as several species move between the main channel, the riparian zone and floodplain water bodies at certain stages of their life cycle (Jungwirth et al. 2000; Schiemer 2000; Schiemer and Waidbacher 1992). In addition, “rhithralisation” effects can also be assumed. As Jungwirth et al. (1995) described, channelization and straightening often caused such a shift to rhithral species due to reduced habitat heterogeneity, higher velocity and coarser bed sediment in the main stem. Tench or weatherfish, though rare in the historical Salzach, were not observed in today’s Austrian rivers. Moreover, the distribution of bream or bleak, as examples for more eurytopic species, is smaller and the relative occurrence decreased.

In addition to the influence of human management, the decrease of soil erosion due to afforestation within the whole catchment is known as one of the main processes affecting the fluvial dynamic of alpine rivers. Reduced bed-load transport induces incision of the river bed, narrowing of the river channel and its disconnection from lateral flood plain river. Finally, such rivers shift naturally from a braided style towards a straightened channel (Pont et al. 2009).

### The effects of log driving

No impact of log driving on fish was determined by our study. This was even true for brown trout, bullhead and grayling for which an adverse effect could have been expected and were usually reported (see e.g. Österreichischer Fischereiverein 1884). Several assumptions help explain our results.

First, only relative abundance information was available which can refer to very different absolute abundances. Also, the statistical test applicable is not very sensitive to the abundance classes. Second, those rivers not used for wood transport in the 1880s were impacted by other human uses, especially hydropower production for mills and other purposes which no doubt adversely affected fish populations. Third, log driving occurred during specific and limited time periods which have to be evaluated in terms of the respective life stage of potentially affected fish. In the Salzach catchment, brown trout was most concerned. Based on studies of the life cycle of trout in the Alps (Crisp 1993; Hari et al. 2006; Schmutz et al. 2013) the start of log driving in late April or May impacted mainly emerging fry and juveniles. Experiments on the influence of hydropeaking, which is somewhat comparable to that of log driving, demonstrated a considerably decreasing impact with each week of growth (Schmutz et al. 2013).

Several studies in Scandinavia, USA and Canada where log driving continued into the 1950s or 1960s prove negative effects on fish, but mainly for the 20th century. For Swedish rivers, Palm (2007) describes the decline of salmonid densities. There, Nilsson et al. (2005) found a decrease of salmon catch in the 1900s and after. This coincided with the most intensive period of log driving, but the authors conclude that it is difficult to assign the declining catches to this pressure explicitly because other factors such as lower fishing effort or hydropower plants and channelization might have played a role as well. Norrgard (2011) pinpointed a decrease of salmon and trout in the Klarälven since the 1850s which was related to intensified log driving management (e.g. systematic removal of boulders). He emphasizes that the low impact of log driving before the 1850s could have reflected special management agreements between wood floating and fishermen. On the Pacific coast of the USA splash dams have been reported in the 1950s to stop the spawning runs of salmonids (Sedell et al. 1991). In a tributary of the Fraser the implementation of new log driving practices in 1963 had particularly negative consequences (Cowell 1981). Apart from the impacts on fish and biota, several studies investigated how historical and recent log driving as well as forestry management altered river morphology (e.g. Wohl 2005; Phelps (2011) citing several other studies; Poux et al. 2011). Clear cutting within short periods influenced in particular in-stream channel structures (woody debris) and sediment budgets (see e.g. Slaymaker 2000 for British Columbia). In contrast to most European countries, where log driving was abandoned in the early 20th century, this activity was often intensified or even only started in USA, Canada or in Scandinavia in that period and after to supply large industries, such as saw mills or pulp and paper industries. From such examples, it appears that, in contrast to the pre-industrial practices in Europe for which the Salzach catchment are representative, modern forest management, including deforestation, had a more negative impact on the ecological functioning of rivers and their fish communities (Welcomme 1985).

## Conclusions

Our case study of the Salzach catchment demonstrates the impact of successive human uses on the long-term change in the fish community, whereby the quality of the historical sources describing the occurrences and relative abundances of fish species within this river network at the turn from the 19th to the 20th century was crucial.

The fish species composition and abundance classes of the Salzach catchment changed throughout the 20th century. This most likely reflected channelization, flood protection, dam erections and species introductions. Pollution or land use change may have played a role as well. Many rheophilic and eurytopic fish species historically inhabited river reaches along an extensive longitudinal profile and were present in more upstream river reaches than today. This was especially true for sensitive species requiring different habitat types during their life cycles (pike, nase, Danube salmon). This decrease of species diversity in the upstream part of river network is a consequence of the lost lateral connectivity because floodplain water bodies or small brooks disappeared and because habitat diversity decreased. These changes in the fluvial dynamics are related not only to the local human management of the rivers but also to the afforestation of catchments and the subsequent decrease of bed load transport and river incision. Furthermore, climate variability significantly impacted the spatial distribution of several fish species (Pont et al. 2015).

One of the most important shifts in the fish community structure is the result of the deliberate introduction of fish species for fisheries. Rainbow trout and brook trout, absent historically, today represent up to 29 % of the total number of fish occurrences. This situation is particularly interesting because, as opposed to most European rivers, rainbow trout is now acclimated and reproduces naturally in Austrian Alpine rivers (Jungwirth et al. 2003).

In contrast, log driving, one of the most common historical pressures in European mountainous rivers, did not show significant negative effects on the historical fish community. This result strongly differs from the severe impacts of log driving and deforestation demonstrated for recent times, and could be related to the change in log driving practices during the 20th century. The high societal value of fisheries before the industrial period may have led to specific management practices. In addition, rivers without log driving were probably affected by other human uses such as the operation of mills.

In general, our results can be valid for a large number of European mountainous rivers, especially in the Alps, and similar rivers and catchments in North America. Even though the Salzach fish community was no longer natural at the end of the 19th century, our reconstruction of the historical situation can serve as a pre-industrial baseline for future restoration in such river types. In the Salzach catchment, several measures are planned in the next 10–15 years to fulfill the requirements of the European Water Framework Directive (BMLFUW 2009). The first step envisions re-establishing the longitudinal connectivity in the main river Salzach. Also, the tributaries are to be reconnected. Further restoration measures encompass the widening of the river bed at least on a local scale (Schmall and Ratschan 2011). These measures can for instance enhance the habitat availability for species such as nase or Danube salmon. Our results highlight the utility of detailed historical studies to understand the long-term evolution of fish communities and their present functioning. Such studies can support river managers in defining targets for the protection and the restoration of freshwater fish biodiversity. Similar research should be done on other types of rivers (e.g. lowland rivers).
